# Mosaicism of XX and XXY cells accounts for high copy number of *Toll like Receptor 7 and8* genes in peripheral blood of men with Rheumatoid Arthritis

**DOI:** 10.1038/s41598-019-49309-4

**Published:** 2019-09-09

**Authors:** Gabriel V. Martin, Sami B. Kanaan, Marie F. Hemon, Doua F. Azzouz, Marina El Haddad, Nathalie Balandraud, Cécile Mignon-Ravix, Christophe Picard, Fanny Arnoux, Marielle Martin, Jean Roudier, Isabelle Auger, Nathalie C. Lambert

**Affiliations:** 1 INSERM UMRs 1097 Arthrites Autoimmunes, Aix Marseille Université, Marseille, France; 2 Arthritis R&D, Neuilly-Sur-Seine, France; 3 Service de Rhumatologie, Hôpital Sainte Marguerite, AP-HM, Marseille, France; 4 Aix Marseille Univ, INSERM, MMG, Marseille, France; 5 Centre National de la Recherche Scientifique (CNRS) UMR7268 (ADES), “Biologie des Groupes Sanguin”, Marseille, France; 6 Etablissement Français du Sang (EFS), Marseille, France

**Keywords:** Immunogenetics, Rheumatoid arthritis

## Abstract

The X chromosome, hemizygous in males, contains numerous genes important to immunological and hormonal function. Alterations in X-linked gene dosage are suspected to contribute to female predominance in autoimmunity. A powerful example of X-linked dosage involvement comes from the *BXSB* murine lupus model, where the duplication of the X-linked *Toll-Like Receptor 7* (*Tlr7)* gene aggravates autoimmunity in male mice. Such alterations are possible in men with autoimmune diseases. Here we showed that a quarter to a third of men with rheumatoid arthritis (RA) had significantly increased copy numbers (CN) of *TLR7* gene and its paralog *TLR8*. Patients with high CN had an upregulated pro-inflammatory JNK/p38 signaling pathway. By fluorescence *in situ* hybridization, we further demonstrated that the increase in X-linked genes CN was due to the presence of an extra X chromosome in some cells. Men with RA had a significant cellular mosaicism of female (46,XX) and/or Klinefelter (47,XXY) cells among male (46,XY) cells, reaching up to 1.4% in peripheral blood. Our results present a new potential trigger for RA in men and opens a new field of investigation particularly relevant for gender-biased autoimmune diseases.

## Introduction

About 80% of patients affected by autoimmune diseases are women^1^. The X chromosome (X Chr) contains numerous genes important to immunological and hormonal function and alterations of X Chr genes and DNA sequences could potentially lead to autoimmunity^2,3^. Genome-wide association studies have been able to identify a number of genetic polymorphisms on the X Chr in association with autoimmunity^4^ but struggle to explain why despite the disadvantage of hemizygosity in men, women are still disproportionately affected. This suggests that X-linked genetic dosage rather than single-nucleotide polymorphisms might be more relevant. Dosage alterations include copy number (CN) variation, estimated to cover 12% of human genome, and representing an important element of genomic polymorphism and population diversity^5^. Interestingly, CN variations are more common in genomic regions containing immunity genes^6^ and, in many cases, are associated with autoimmune diseases^7^. Chromosome monosomy or trisomy can also account for gene dosage alterations.

In women, Turner’s syndrome occurs in about 1:3,000 to 1:2,500 live-born girls where half of them would acquire full X monosomy (defined by total or partial absence of one X Chr in all cells) while the other half would have mosaic X monosomy (i.e. some cell lineages with 45,X others with 46,XX)^8^. Low-frequency mosaicism of 45,X cells can go unnoticed as women can have a normal reproductive lifespan and be phenotypically normal^9^. Mosaic X Chr aneuploidy is frequently observed in T-lymphocytes in aged women and a positive correlation between quantities of 45,X cells and advancing age in women has been demonstrated by Russel *et al*.^10^. Of incidental note, age-related loss may be tissue specific as this can occur in peripheral blood lymphocytes while it is rarely observed in bone marrow^10^. X Chr trisomy (47,XXX) is also relatively common (~1 in 1,000 live female birth) and is accompanied with increased prevalence of some autoimmune diseases in these women^11^.

In men, the most common numerical chromosomal aberration is Klinefelter syndrome (47, XXY), affecting 1:1,000 to 1:500 live-born boys. Moreover, underdiagnosed ‘*mosaic* Klinefelter syndrome’, where as low as 2% of cells could show a 47,XXY karyotype in a regular 46,XY background, can also occur^12,13^. Another source of supernumerary X Chr in men could come from the presence of maternal cells (46,XX) naturally acquired during *in utero* life and persisting in the growing child and adult^14^. This phenomenon, called maternal microchimerism (Mc), has been well documented in association with many autoimmune diseases^15,16^.

A powerful example of X-linked dosage involvement in autoimmunity comes from the BXSB murine lupus strain, where an unbalanced translocation of a 17-gene cluster from the X to the Y Chr has been shown to be responsible for accelerating pathogenesis of autoimmunity only in males^17^. This translocation called Y-linked autoimmune accelerator (Yaa) includes innate immunity genes *Tlr7* and *Tlr8*, coding respectively for Toll-like receptor (TLR) 7 and TLR8 proteins. The duplication of *Tlr7* is demonstrated to be required to accelerate autoimmunity in lupus susceptible male mice^18^.

TLRs are evolutionarily conserved innate immune proteins and are critical in first-line defense against foreign agents. TLR7 and TLR8 are located in endosomal compartments and both recognize single-stranded RNA^19^. *TLR7* and *TLR8* genes are phylogenetically related as they are paralogs. They are located at close proximity of each other in a region of the X Chr syntenic in humans and mice, on the short arm of the X (Xp22.2).

*Tlr7* duplication and translocation in the Yaa mouse model reveals the importance of supplementary genetic material from the X Chr in triggering autoimmunity. X-linked gene duplications, similar to that of the Yaa mouse model, could happen in humans as CN variations are a common phenomenon^5^ and could activate autoimmunity in men as it does in male mice. This hypothesis was first tested in an American cohort of males and females with systemic lupus erythematosus (SLE), a female-predominant autoimmune disease, but did not show a significant increase in *TLR7* CN in patients^20^. Nevertheless, a larger study in a Mexican cohort of childhood-onset SLE showed a significant increase in *TLR7* CN associated with the disease, and particularly in males^21^. Finally a Chinese study analyzed CN variations of multiple *TLR* genes and found that the X-linked *TLR7*, but not the autosomal *TLR9* gene, was increased in CN in Behçet’s disease, a chronic auto-inflammatory disorder^21,22^. In contrast to the Yaa model, the increase of *TLR7* CN in both the Mexican and Chinese studies was partial, i.e. not a duplication in all cells, and its origin was not explained.

To see whether such X-linked alterations can be further generalized in autoimmunity, rheumatoid arthritis (RA), a female-predominant chronic autoimmune disease of the synovial joints, was studied. In a pilot analysis, we had tested on a small number of DNA samples from men with RA the hypothesis that CN of the *TLR7* gene, as well as its neighboring paralog *TLR8*, was increased compared to healthy men^23^. We had showed a significant CN increase of both *TLR* genes in peripheral blood mononuclear cells (PBMCs) from men with RA when compared to healthy men by real-time quantitative PCR (qPCR) assay using an autosomal housekeeping gene as reference.

In the current study, we optimized and validated qPCR assays with a second reference gene and on a large number of study participants, with an extended age-range from birth to 82 years old. We further investigated whether increased CN of *TLR7* and *TLR8* influences *TLR7/8* mRNA levels and mRNAs of proteins involved in TLR pathways. Finally, we demonstrated the origin of *TLR7/8* CN variation by Fluorescence *in situ* Hybridization (FISH) on nuclei in metaphase and interphase from men with RA and healthy men.

## Results

### Validation of *TLR7* and *TLR8* CN assessment method on healthy controls

**Figure 1:**
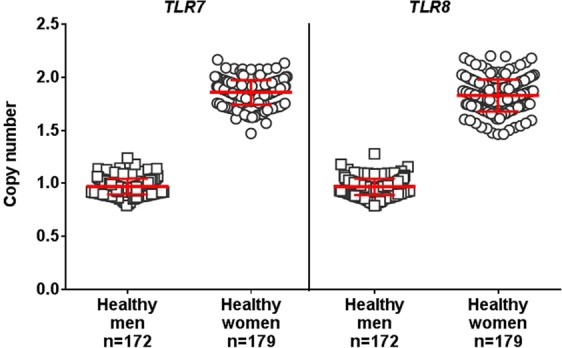
Validation of *TLR7* and *TLR8* copy number assessment method on healthy individuals. *TLR7* and *TLR8* gene copy numbers (CN) were calculated by comparison with the mean of two reference genes, as detailed in the method section, on peripheral blood DNA samples from 172 healthy men and 179 healthy women. White squares represent individual men and white circles represent women. On each plots, red bars represent mean values and standard deviations.

**Figure 2:**
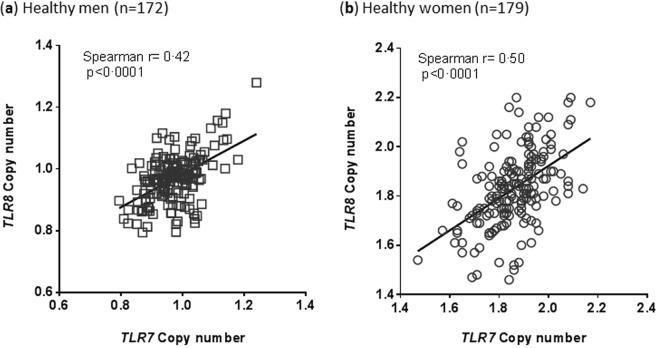
Significant correlation between *TLR7* CN and *TLR8* CN in healthy individuals. (**a**) The same samples from 172 healthy men and (**b**) 179 healthy women were simultaneously tested for *TLR7* and *TLR8* CN. A significant correlation is observed across samples by Spearman’s rank test (*P* < 0.0001).

Peripheral blood DNA samples from 172 healthy men and 179 healthy women were tested for X-linked *TLR7* and *TLR8* CN (Fig. 1). As expected, healthy men had ~1 copy of *TLR7* and *TLR8* (mean ± standard deviation: 0.97 ± 0.07 and 0.97 ± 0.08 copies, respectively) and healthy women had ~2 copies of *TLR7* or *TLR8* (respectively, 1.86 ± 0.12 and 1.83 ± 0.15 copies). Confidence in the precision of qPCR measurements was given by a DNA sample from a healthy man, systematically run as a calibrator, giving similar results for both genes through 32 runs of 384-well plates (*TLR7*: 1.01 copies ±0.04 and *TLR8*: 0.99 ± 0.06, data not shown). Moreover, a strong correlation between *TLR7* CN and *TLR8* CN was seen within each sample from healthy men or healthy women (respectively, Spearman *r* = 0.40 and *r* = 0.50, *P* < 0.0001, Fig. 2).

### Increased *TLR7* and *TLR8* gene CN in blood samples from men with RA

**Figure 3:**
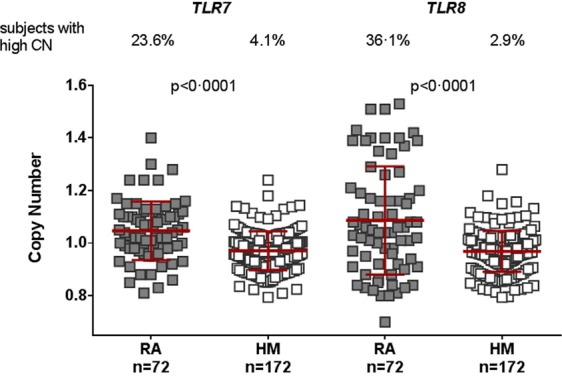
Increased copy number of *TLR7* and *TLR8* genes in blood samples from men with RA. *TLR7* and *TLR8* gene copy numbers (CN) were calculated as detailed in the method section on peripheral blood DNA samples from 64 men with RA (RA) compared to 171 healthy men (HM). *P* values are representative of Mann-Whitney test and indicate quantitative differences in CN between men with RA and HM. Grey filled squares represent individual men with RA and unfilled squares represent HM. On each plots, red bars represent mean values and standard deviations. High CN of *TLR7* and *TLR8* genes was defined as having a z-score ≥2, i.e. being superior or equal to the mean value observed in HM plus two standard deviations (≥1.11 for *TLR7* CN and ≥1.13 for *TLR8* CN represented by the blue dot line). Percentages of men with high CN are indicated in the upper section of the figure.

Using the validated qPCR assays, we found that men with RA had significantly increased *TLR7* CN (mean: 1.05 ± 0.11 copies) and *TLR8* CN (mean 1.09 ± 0.21 copies) in blood DNA samples compared to healthy men (respectively, 0.97 ± 0.07 copies, *P* < 0.0001 and 0.97 ± 0.08 copies, *P* < 0.0001, Fig. 3). We defined an individual with “high CN” as having a CN value with a z-score ≥2, i.e. being superior or equal to two standard deviations above the mean value observed in the healthy men (≥1.11 for *TLR7* and ≥ 1.13 for *TLR8* CN), thus, high CN is associated with a confidence level of ≥97.72%. With this threshold, 24% of men with RA had high *TLR7* CN compared to only 4% of healthy men (*P* < 10^−6^, χ^2^ test). Similarly 36% of men with RA had high *TLR8* CN compared to only 3% of healthy men (*P* < 10^−11^, χ^2^ test). Unlike the 2-fold increase in the BXSB mouse model, men with RA with high CN had a mean of 1.20 *TLR7* copies (not shown on the graph) which, assuming a duplication, corresponds to about 8% of cells with 2 copies when compared to the 1.11 threshold (at z-score = 2).

Together, our data suggest that *TLR7* and its neighboring paralog *TLR8* (or the genetic region containing them) are at increased CN in peripheral blood of RA men compared to healthy men.

### *TLR7* and *TLR8* CN did not increase with age in healthy controls

**Figure 4:**
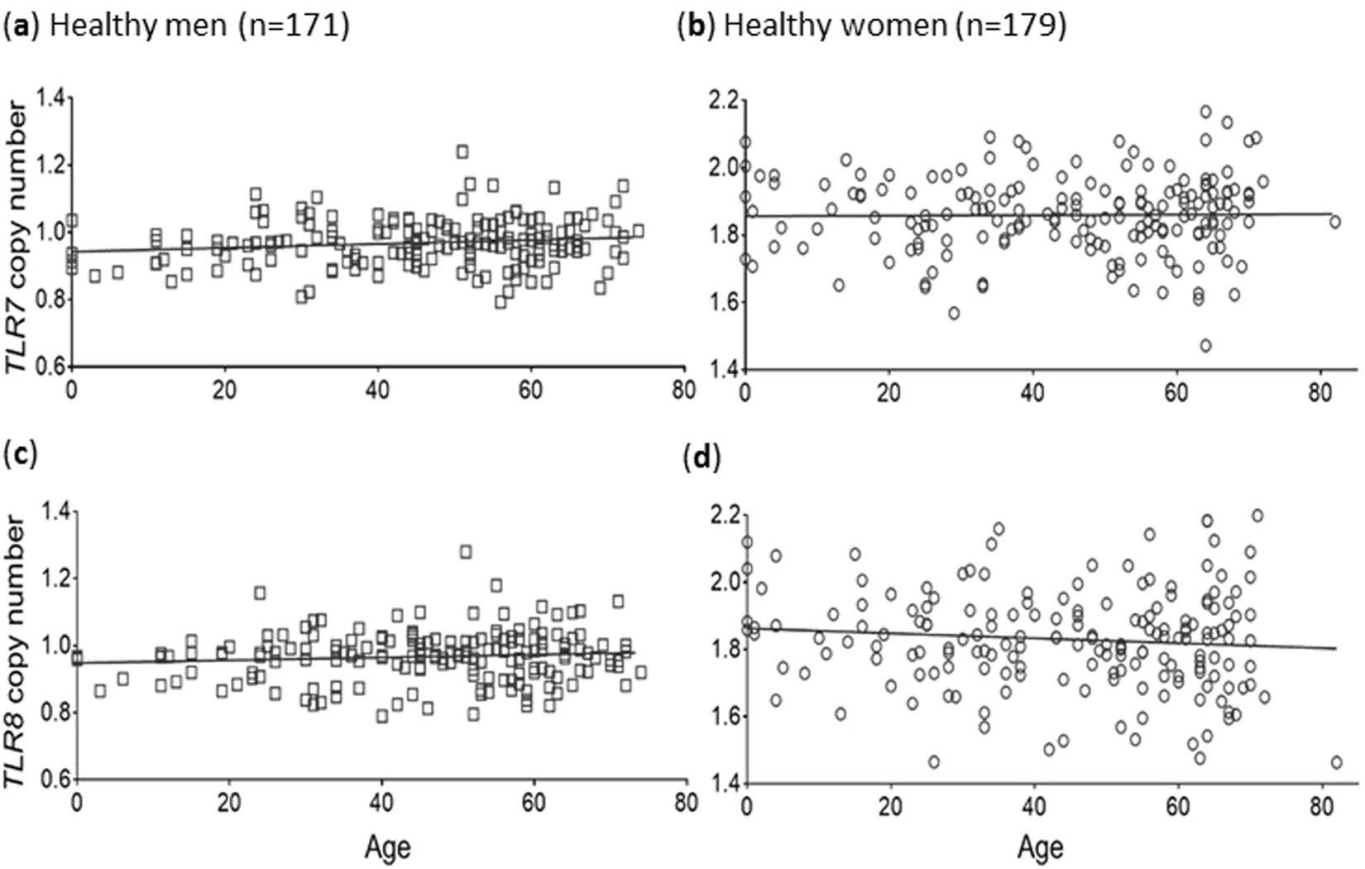
No influence of age in *TLR7* and *TLR8* CN in healthy individuals. *TLR7* CN was evaluated on peripheral blood DNA samples from (**a**) 171 healthy men and (**b**) 179 healthy women from birth to 74 years old and birth to 82 years old. *TLR8* CN was evaluated on same samples from (**c**) 171 healthy men and (**d**) 179 healthy women. Spearman correlation test was not significant for any of the graphs.

Because the incidence of RA in men rises steeply with age, and the accumulation of somatic duplications with age is a possibility, we evaluated the influence of age on *TLR7*/*8* CN. *TLR7* and *TLR8* CN did not increase with age in either DNA samples from peripheral blood of healthy men from birth to 74 years old or healthy women from birth to 82 years old (Fig. 4).

*TLR7* and *TLR8* CN were similarly significantly increased in men with RA compared to healthy men when men from both groups were age-matched (Suppl. Fig. S1).

### No particular cell subpopulation being at increased *TLR7* or *TLR8* CN

Because the significant CN increase observed in blood samples from RA patients was not a 2-fold increase and corresponded to only a small percentage of cells from peripheral blood having more than 1 copy of *TLR7/8* genes, we asked whether a particular cell subpopulation undergoing, for example, clonal expansion could be affected by this gene CN variation. When analyzing DNA samples from PBMC instead of DNA samples from whole blood, increased *TLR7/8* CN was still significant in men with RA compared to healthy men (Suppl. Fig. S2). However, when analyzing in regard to specific cell populations such as B cells, T cells, granulocytes or the depleted fraction of the former three, no specific cell subpopulation was at increased CN (Suppl. Fig. S3), suggesting any cell type could be affected.

### TLR-signaling pathway genes are differently regulated in PBMC from RA patients with high *TLR7/8* CN compared to normal CN

**Figure 5:**
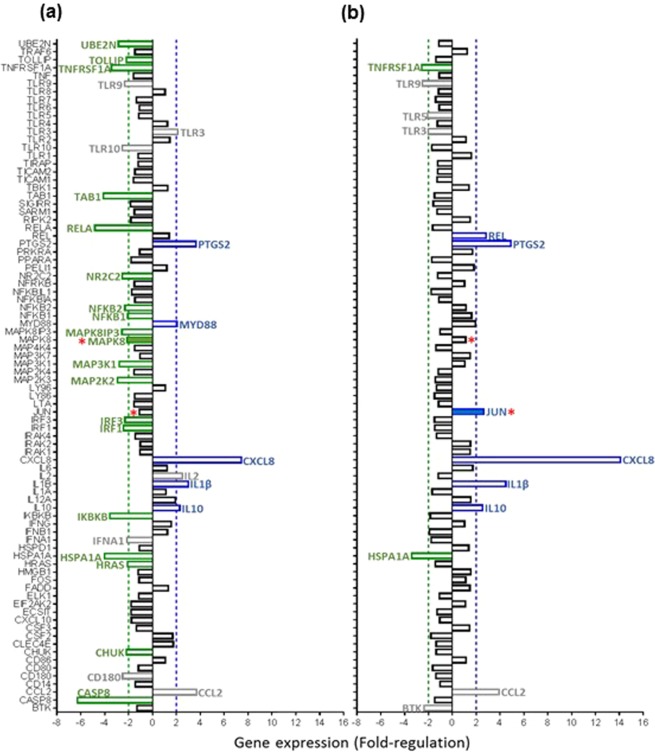
Expression profile of 84 genes involved in the TLR signaling pathway in men with RA according to their *TLR7* CN. PBMC mRNA transcript levels of 84 genes involved in the TLR signalling pathway were measured (**a**) for 6 men with RA with *TLR7* CN < 1.11 called group A and (**b**) for 9 men with RA with *TLR7* CN ≥ 1.11 called group B, each group compared to data from 14 healthy men. Gene expression was realized by reverse transcription qPCR using RT² profiler PCR array and data represented in fold regulation. Fold-Change (2^−ΔΔCt^) was the normalized gene expression (2^−ΔCt^) in the RA samples divided by the normalized gene expression (2^−ΔCt^) in the control samples. Fold-Regulation represented fold-change results in a biologically meaningful way. Significantly upregulated genes compared to healthy controls presenting fold-regulation values greater than 2 are indicated in blue. Significantly down-regulated genes compared to healthy controls presenting fold-regulation values lower than −2 are indicated in green. Genes noted in grey are not considered for statistical analysis as they have an average threshold cycle relatively high (>30), meaning that their relative expression level is low, in both control and patient samples, and the p-value for the fold-change is either unavailable or relatively high (*P* > 0.05). Genes for which mRNA expression is significantly different between the 2 groups of RA patients (group A with a normal *TLR7* CN and group B with a high *TLR7* CN) are noted with a red asterisk (respectively MAPK8, *P* = *0.049* and JUN*, P* = *0.036*).

We then asked whether *TLR7/8* CN increase in men with RA had any influence on *TLR7* and *TLR8* mRNA production and consequent TLR signaling pathway. The expression of mRNA from 84 genes related to TLR-mediated signal transduction, including the 10 *TLR* genes, was evaluated by RT-qPCR in PBMC obtained from 15 men with RA and 14 healthy men. Men with RA were divided into 2 groups according to *TLR7* CN results by qPCR (Fig. 5), a group A of 6 men with normal *TLR7* CN (<1.11) (Fig. 5a) and a group B of 9 men with high *TLR7* CN (≥1.11) (Fig. 5b). Data from both groups were referred to results from healthy men. Patients with high *TLR7* CN (group B) did not show higher *TLR7* or *TLR8* mRNA expression relative to patients with normal *TLR7* CN (group A).

Nevertheless the 2 groups had a different gene expression profile. In the group A, a total of 19 genes were down regulated (in green Fig. 5a), with one of them being inversely and statistically up-regulated in group B: *Mitogen-Activated Protein Kinase 8* (*MAPK8*, *P* = 0.049, Fig. 5a,b). Five genes were up-regulated in the group A: *Prostaglandin-endoperoxide synthase 2* (*PTGS2*), *Myeloid differentiation primary response gene 88* (*MYD88*), *Interleukin 8* (*CXCL8*), *Interleukin 1β* (*IL1β*), and *Interleukin 10* (*IL10*) (in blue Fig. 5a). All of them were also up-regulated in the group B with a greater, but not significant, up-regulation of *CXCL8* in the group B (Fig. 5b). In the group B, only two genes were down-regulated (in green Fig. 5b): *Heat shock 70* *kDa protein 1* *A* (*HSPA1A*) and *Tumor necrosis factor receptor superfamily, member 1* *A (TNFRSF1*) but were similarly down regulated in group A. Among the six genes up regulated in the group B (in blue Fig. 5b), only *Jun proto-oncogene* (*JUN*) was significantly upregulated compared to patients with a normal CN (*P* = 0.036).

Together, our data show unchanged *TLR7* and *TLR8* mRNA levels between patients with high *TLR7* CN compared to men with ‘normal’ *TLR7* CN. Nevertheless, a different TLR-linked mRNA expression profile is observed with a significant up-expression of *MAPK8* and *JUN* mRNA in patients with high *TLR7* CN.

### XX and XXY mosaicism among XY cells

**Figure 6:**
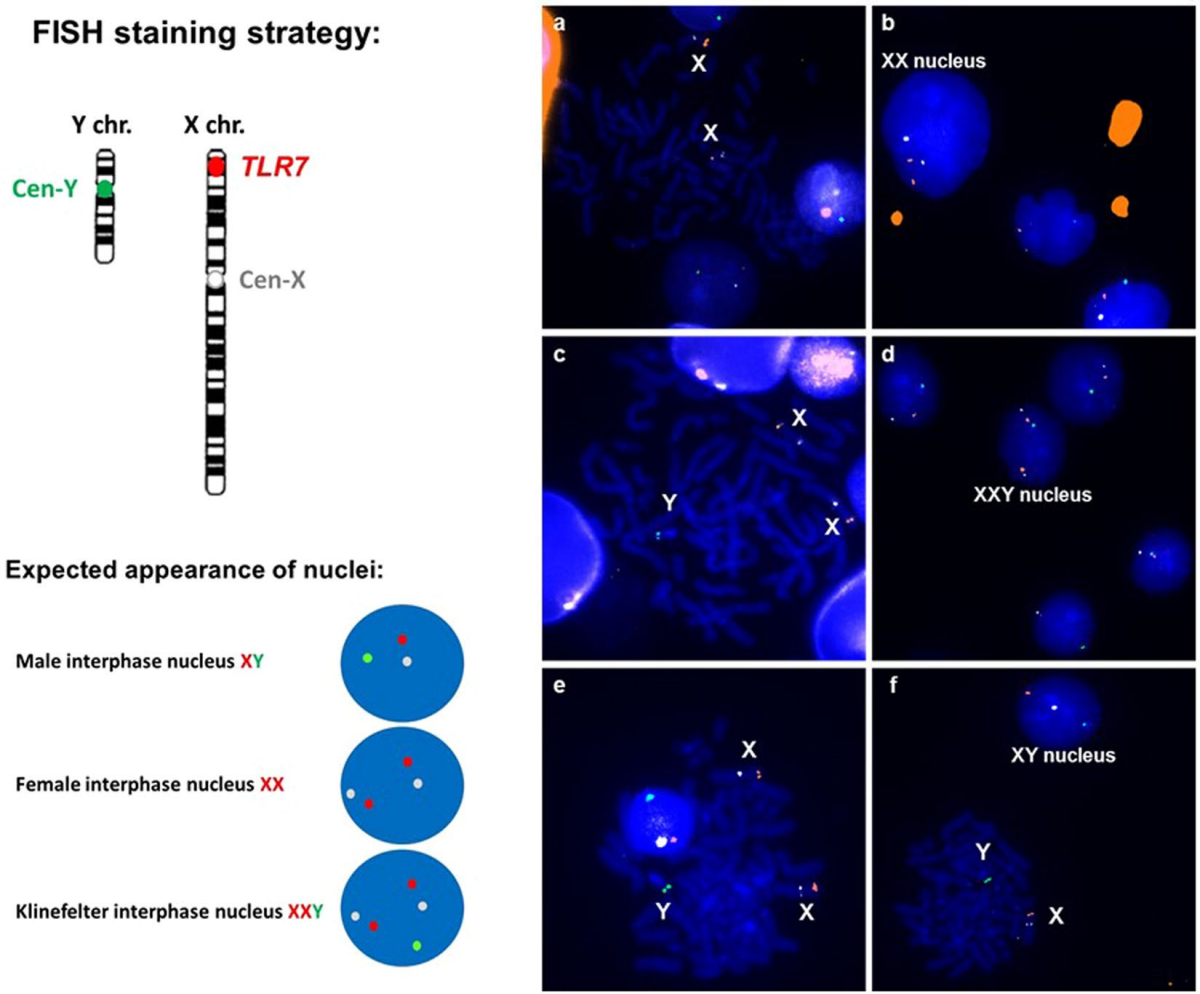
Presence of XX and XXY cells in subjects with high CN of *TLR7* and *TLR8* genes. FISH was conducted on nuclei in interphase and metaphase from peripheral blood cells of 9 men with RA and 8 healthy men. Nuclei were hybridized with an orange 5′ TAMRA-*TLR7* probe, an aqua X-centromere specific probe and a green 5′ Fluorescein Y-centromere specific probe. All signals were in the same plane of focus. The aqua channel (X centromeric probe) was digitally changed to white colour in the imagery software Zen 2 to avoid its confusion with the green fluorescence (optical magnification x63). Images are FISH representations from study subjects: three men with RA and one healthy control. Presence of XX cells (**a**) in metaphase of RA patient #1, (**b**) in interphase of RA patient # 2. Presence of XXY cells, (**c**) in metaphase of RA patient #1, (**d**) in interphase of RA patient # 3, (**e**) in interphase of healthy control # 1. Male nucleus representative of any other male nuclei observed in men with RA or healthy men is represented in (**f**) male nucleus (46, XY) in interphase of healthy control # 1. Images were visualized on a Zeiss AxioImager Apotom Z1 fluorescent microscope and an AxioCam MRm camera (Zeiss, Jena, Germany).

Next, we investigated whether the origin of the increased *TLR7/8* CN was due to the duplication of *TLR7/8* region (with or without translocation) or to an extra X Chr in some cells. FISH was performed with two X Chr-specific probes (X centromere and *TLR7*) and one Y centromere-specific probe on peripheral blood cells in metaphase from 14 men with RA (5 with normal CN and 9 with high CN) and 11 healthy men (6 with normal CN and 5 with high CN) (Fig. 6). FISH results did not show a *TLR7* duplication, rather the presence of XX nuclei (Fig. 6a,b) and XXY nuclei (Fig. 6c–e) among normal XY male nuclei (Fig. 6f). A mean of 1,547 nuclei were analyzed per sample (range [1,103–3,456]) and the number of XX or XXY nuclei were reported per 10,000 nuclei.

### qPCR results correlated with data from FISH analyses

**Figure 7:**
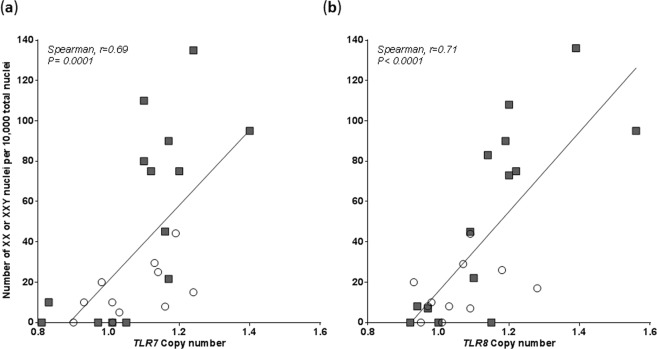
(**a**) Copy number of *TLR7* gene or (**b**) *TLR8* gene determined by qPCR correlates with the number of nuclei with 2 X chromosomes (46, XX or 47, XXY cells) found by FISH in the same sample. Results are from 14 men with RA (grey squares) and 11 healthy men (unfilled circles) tested for both methods.

We found that men who had the highest number of nuclei with an extra X Chr by FISH had the highest *TLR7 CN* (Fig. 7a) *or TLR8* CN (Fig. 7b) by qPCR (respectively Spearman, r = 0.77 and r = 0.71, *P* < 0.0001). Overall men with the highest *TLR7/8* CN had a higher number of XXY nuclei rather than XX nuclei (see individual repartition of samples tested by FISH in Suppl. Fig. S4).

There was a stronger correlation between *TLR7/8* CN and the number of Klinefelter XXY nuclei observed compared with the number of female XX nuclei, yet the correlation was statistically significant in all cases (Suppl. Fig. S5).

Together, these data suggest that *TLR7/8* CN increase is not due to genomic duplications but rather as a consequence of the presence of extra X chromosomes, particularly XXY cells.

## Discussion

In the current study, we have presented evidence, on a large number of individuals, that the copy number of X-linked *TLR7* and *TLR8* genes are increased in peripheral blood cells of men with RA compared to healthy men. Because the incidence of RA in men rises steeply with age^24,25^, because men with RA from our cohort are significantly older than healthy donors and somatic duplications could accumulate with age, we evaluated the incidence of age on *TLR7/8* CN variation. As evidenced by results on 351 healthy individuals from birth to 82 years old, *TLR7/8* CN variation is not age-dependent. Nevertheless, unlike the 2-fold increase in the BXSB mouse model, CN increase represents a small percentage of cells having more than 1 copy of *TLR7/8* genes in a quarter to a third of men with RA. To see whether this increase could be restricted to a subpopulation of cells undergoing e.g. clonal expansion, we further analyzed cell subsets. No particular cell subpopulation (B cells, T cells, granulocytes, or others) had an increased *TLR7/8* CN, thus rejecting this hypothesis and suggesting that all cell types could be similarly affected.

We further investigated the consequences of this increased CN at the mRNA level. *TLR7* and *TLR8* mRNA expression was not increased in men with high *TLR7/8* CN as it would have been if *TLR7/8* genes were duplicated and translocated on another chromosome, as described in the Yaa mouse model. Nevertheless, we showed that men with RA with high *TLR7/8* CN had a different TLR signaling pathway profile than men with RA with normal CN. In general, men with high CN had less down regulated genes than men with normal CN. Both group of patients had a strong *CXCL8* (*IL-8*) up-regulation with a greater expression in patients with high CN. This cytokine has previously been described with higher levels in synovial fluid from RA patients compared with synovial fluid from osteoarthritis patients^26^. Finally, mRNA expression of *MAPK8* and *JUN* was significantly increased in patients with high CN when compared to patients with normal CN. Interestingly, both genes are involved in the JNK/p38 signaling pathway, a pro-inflammatory pathway already known to be up regulated in RA^27^. Furthermore MAPK8 is involved in IL-8 expression in synovial fibroblasts^28^.

The increased copy number of *TLR7/8* without increased levels of *TLR7/8* mRNA suggested it was due to the presence of two X Chr in some cells as seen in Klinefelter cells (XXY) cells or female cells (XX). In both cases, one of the two Xs would be randomly inactivated and thus *TLR7/8* mRNA levels unchanged despite an increase in the copy number of the involved genes. Female (46,XX) and Klinefelter (47,XXY) nuclei were indeed found by FISH among normal male (XY) nuclei among the 25 men (14 RA and 11 healthy) we could test. Importantly, samples giving high quantity of cells with a supernumerary X Chr by FISH were samples giving high *TLR7/8* CN by qPCR. The correlation with qPCR results was stronger with XXY cells rather than with XX cells (*P* < 0.0001 versus *P* = 0.01). XX and XXY mosaicism was particularly high in blood samples from patients with RA.

While uncommon, Klinefelter syndrome, has been associated with increased risk of developing some autoimmune diseases, including RA^29^. Here we demonstrate the presence by FISH of less than 1% of 47,XXY cells among 46,XY background cells; this could be the result of ‘mosaic Klinefelter syndrome’ that generally goes undiagnosed, as mosaic Klinefelter men often lack symptoms^13^. Nevertheless, the supernumerary X chromosome in XXY cells can still contribute to immunological disorders as this chromosome carries a large percentage of genes linked to immunity. A recent study shows that the Klinefelter syndrome is associated with high recurrence of CN variation on the X chromosome and particularly duplications^30^. It is very possible that, similar to what is observed in Klinefelter syndrome, XXY cells from men with RA, are prone to X-linked gene duplication. If a *TLR7/8* duplication occurs with translocation very close to the original gene that would go unnoticed by FISH. This would then explain why the correlation between CN obtained by qPCR and number of cells with a supernumerary X Chr obtained by FISH was significantly higher with XXY cells than with XX cells.

Maternal Mc could contribute to a gain of female cells (46,XX nuclei) in men with RA, explaining a gain in *TLR7/8* copies. Most patients did not have a mother alive or willing to participate, therefore we could test only four of them for maternal Mc as well as six healthy men (data not shown). All samples positive for maternal Mc by non-inherited maternal HLA specific qPCR (as previously described^31^) were found positive by FISH for female cells, and all samples negative by FISH for female cells were always negative for maternal Mc by HLA-specific qPCR. Nevertheless, the number of subjects studied for maternal Mc was too limited to draw definite conclusions, but results seemed to support for the most part, the maternal origin of the XX nuclei. Other sources could be from a female twin, including a vanished twin, as we previously described in a man with a scleroderma-like disease^32^, older female sibling or prior maternal miscarriage. Lack of availability of other family members prohibited exploring these possibilities further in the current study.

It is to be noted that by FISH we found at best 1.4% of cells with a supernumerary X Chr, while by *TLR7/8* qPCR men with RA who had high *TLR7* CN had a mean of 1.20 copies compared to men with normal CN, which corresponds to about 8% of cells with 2 copies (cf results). This percentage difference is a recurrent difference of sensitivity between the two methods. FISH is known to be less sensitive than qPCR with respectively of 0.1% versus 0.005%^32^. Despite the lack of sensitivity, the FISH method was markedly informative for visualizing the origin of increased CN observed in men with RA.

Although men with RA more frequently had a supernumerary X Chr, we did not show an increased *TLR7/8* mRNA expression, suggesting that these genes, at least in PBMC, are normally regulated by X Chr inactivation (XCI), a dosage compensation mechanism used by mammals to ensure that XX females and XY males have similar X Chr gene expression^33^. Nevertheless about 15% of X genes escape XCI^34^ and thus are bi-allelically expressed. Wang *et al*. showed that the XCI is not maintained with the same stability in female lymphocytes than other somatic cells^35^. The inactivated X becomes partially reactivated and single-cell RNA FISH analysis of female T cells revealed that the X-linked genes *CD40LG* and *CXCR3* were bi-allelically expressed in some cells^35^. Similarly Syrett *et al*. observed dynamic chromatic changes on the inactive X allowing B cells during their development to reactivate X-linked immunity genes^36,37^. Furthermore, Souyris *et al*. showed that *TLR7* can escape from XCI in subsets of B cells, monocytes and plasmacytoid dendritic cells from women with SLE and in men with Klinefelter syndrome^38^. X inactivation can vary from one tissue or one cell type to another^39^. Here, we have only analyzed peripheral blood cells and this is very possible that *TLR7/8* genes escape X inactivation in tissue cells such as synovial cells. Moreover it has been shown that in Klinefelter syndrome, the X inactivation process is less effective, as methylation on the X is decreased compared to normal female samples^40^. Other genes on the X Chr with critical immune-related functions beyond *TLR7* and *TLR8*, e.g. *Forkhead Box P3 (FOXP3)*, might have their expression affected by the XXY and XX mosaicism but were not tested in the present study due to limited availability of biological specimens. These will be subject to future investigations that would also study XCI pattern in these mosaic cells.

In conclusion, we have found an increased *TLR7/8* CN in men with RA. This increase is associated with the presence of 46,XX and 47,XXY cells. Men with RA with high CN, i.e. carrying higher amounts of these cells, have an upregulation of genes involved in the TLR signaling pathway, particularly the JNK/p38 signaling pathway. This suggests that the mere presence of a supernumerary X Chr can have functional consequences. Previously, an increased CN of *TLR7* gene has been described in patients with SLE and in patients with ocular Behçet’s disease, with no explanation of the derivation^21,22^. We are the first to present an explanation for the origin of the increase and suggest further investigations in men with other gender-biased autoimmune diseases. The current study gives new insights into the etiology of Rheumatoid Arthritis and opens a new field of investigation particularly relevant for gender-biased autoimmune diseases.

## Patients and Methods

### Study subjects

*TLR7* and *TLR8* CN variations were studied in DNA from 72 men with RA and 172 healthy men and 179 healthy women, all Caucasians. All patients with RA satisfied the 2010 revised criteria of the American College of Rheumatology and the European League Against Rheumatism^41^ and were anti-citrullinated protein antibody-positive (ACPA+). Out of the 72 men with RA studied, we had treatment information for 55 of them, 70.9% were receiving anti-TNF treatment (51.3% Etanercept, 10.3% Infliximab, 12.8% Adalimumab, 2.6% Golimumab). Median age at the onset of RA was 48. Healthy controls had no history of autoimmune disease in the family. To test age influence on CN variation, we recruited healthy males from birth to 79 years old and healthy females from birth to 82 years old.

### Study approval

The study has received the approval of the ethics committee (CPP Sud-Méditerranée II) and is registered at the INSERM (Biomedical Research Protocol RBM-04-10) or as a collection (DC-2008-327). All participants signed informed consent according to the Declaration of Helsinki^42^. All experiments were performed in accordance with relevant guidelines and regulations.

### Cell sorting from whole blood

Heparin lithium anti-coagulated blood was processed by gradient centrifugation (Histopaque 1077, Sigma-Aldrich, MO, USA) to isolate peripheral blood mononuclear cells (PBMC). For some samples, cells were separated with immuno-magnetic cell sorting (RoboSep™, STEMCELL™ Technologies, Canada) into CD19+ (B cells), CD3+ (T cells), CD66b+ (granulocytes) and CD19−/CD3−/CD66b− (monocytes, macrophages, NK cells and dendritic cells). Fractions were checked for purity by flow cytometry with the MACSQuant® device (Miltenyi Biotec, Germany), using CD20-VioBlue®; CD4-(VIT4)-FITC; CD8-PE and CD66abce-APC fluorescent antibodies, following manufacturer’s recommendation. Cell fractions with purity higher than 95% were kept for further analysis.

### DNA isolation

DNA from 350 µL of whole blood was extracted with EZ1 DNA Blood Kit (Qiagen, Germany) using a BioRobot EZ1 system (Qiagen, Germany) according to the manufacturer’s instructions and stored at –20 °C for qPCR assays. DNA from PBMC and/or sorted fractions was similarly extracted, with EZ1 DNA Tissue Kit (Qiagen, Germany), and stored.

### *TLR7* and *TLR8* copy number estimation

A TaqMan® real-time qPCR assay was developed to calculate the *TLR7* or *TLR8* (*TLR7/8*) gene CN variation, using LC480 Probe Master reaction kits (Roche Diagnostics GmbH, Germany) on a LightCycler®480 instrument. All DNA samples (25–35 ng) were tested in triplicate in a final volume of 10 µL. Each sample was simultaneously amplified with a set of primers/probe designed either for *TLR7* or *TLR8* and 2 reference genes: *HBB* (part of the *β-globin* locus) and *RPP30* (gene coding for the Ribonuclease P/MRP 30 kDa Subunit protein). Details on oligonucleotides and thermal cycling conditions are given in Table 1. Data were analyzed using LightCycler®480 software version 1.5. DNA sample from a healthy man was systematically run as a calibrator in each plate. The absolute quantification of copies for each gene was calculated according to their respective standard curves. Standard curves were obtained by two-fold serial dilutions of the calibrator DNA sample (66–4.125 ng). *TLR7* or *TLR8* gene CN was calculated as indicated in the following formula:

$${TLR7}\,{\rm{or}}\,{TLR8}\,{\rm{CN}}=\frac{{absolute}\,{quantity}\,{of}\,{TLR7}\,{or}\,{TLR8}\,}{({absolute}\,{quantity}\,{of}\,{mean}\,(\mathrm{HBB}+\mathrm{RPP30}))\div{2}}$$

**Table 1:** Oligonucleotides used to determine relative *TLR7* and *TLR8* copy numbers by real-time quantitative PCR assays.

Oligonucleotide	Sequence
TLR7 Forward	5′-CAGTATTGTGCTGTCTTTGAAATGTA-3′
TLR7 Reverse	5′-TGGTTGAAGAGAGCAGAGCA-3′
TLR7 Probe	5′-(FAM)TTGGGCCCATCTCAAGCTGATCTTG(TAMRA)-3′
TLR8 Forward	5′-GTGAGGCCACACAAGATGGA-3′
TLR8 Reverse	5′-TTCCAGACCACTCCCTTTGC-3′
TLR8 Probe	5′-(FAM)CGCCCAAGTGTCCACCTAAACATGAGT(TAMRA)-3′
HBD Forward	5′-AGATTCCTACTTTCAGCGTTGG-3′
HBD Reverse	5′-CAGCAGGGTTCAGGAAGATAAA-3′
HBD Probe	5′-(FAM)CAACCTGGATCCACTTGCCCAGTG(TAMRA)-3′
RPP30 Forward	5′-TTGTCGTTCAGAAGAAGACAAAGA-3’
RPP30 Reverse	5′-AGTTGACTAGGGATTCGGAGAAA-3′
RPP30 Probe	5′-(FAM)TGTTGATTTCAACACACAAATTCTGGTGG(TAMRA)-3′

Thermal cycling conditions were 95 °C for 10 minutes followed by 40 cycles at 95 °C for 15 seconds and 60 °C for 60 seconds.

### Extraction of RNA and cDNA synthesis

RNA was extracted from 3 to 5 million −80 °C frozen PBMC using the RNeasy® Plus Mini Kit (Qiagen, Germany), cDNA synthesis was realized using RT^2^ Pre AMP cDNA Synthesis Kit (Qiagen, USA) according to the manufacturer’s protocols. RNA quality was analyzed on a Biodrop and met the required criteria for RT-PCR arrays.

### Gene expression of the TLR signaling pathway

The Human Toll-Like Receptor Signaling Pathway RT² Profiler PCR array (SABiosciences) was used to profile the mRNA expression of 84 genes related to TLR-mediated signal transduction (Suppl. Table S1). Negative control for genomic DNA and contaminating RNA were also conducted in each experiment. Amplification, data acquisition, and the melting curve were carried out by the LightCycler®480 instrument (Roche Diagnostics GmbH, Germany). The PCR cycling program was set up according to the manufacturer’s instructions. The fold-change of each gene of interest compared to the control group was calculated as 2^−ΔΔCt^ using *B2M* and *ACTB* as housekeeping genes. Data were analyzed using RT² profiler PCR Array Data Analysis version 3.5 (SABiosciences).

### Metaphase of peripheral blood leukocytes

Cells from 600 µL of peripheral blood from 14 men with RA and 11 healthy men were cultured in 25 mL flasks (NUNCLON^TM^ ΔSurface, Nunc^TM^ Brand Products, Denmark) with 5 mL of Roswell Park Memorial Institute Medium (RPMI, Lonza, Switzerland) supplemented with 15% of fetal bovine serum (FBS, Eurobio, France), 50 µL of L-glutamine (200 mM, Lonza, Switzerland), 50 µL of penicillin – streptomycin (10 mg/ml, Sigma-Aldrich, France), 100 µL of PhytoHemagglutin A (Eurobio, France) and incubated 72 hours (mitotic activity peak with 45% of cells in S-phase) at 37 °C, 5% CO2. Then, 44 µL of fluoro-deoxyuridine and 175 µL of uridine (0.1 mM, Sigma-Aldrich, France) were added to the culture to stop the cell cycle at the S-Phase. Cell culture was then further incubated 15 hours at 37 °C, 5% CO2. To release the S-Phase block and allow cell culture synchronization 250 µL of bromo-deoxyuridine (3 mg/ml, Sigma-Aldrich, France) were added and further incubated 7 hours at 37 °C, 5% CO2. To disrupt the mitotic spindle and inhibit cell division 50 µL of colchicine solution (20 mg/ml, Eurobio, France) were added. After 1.5 hours cells were harvested in a 15 mL tube and centrifuged 10 minutes at 1100 rpm. A hypotonic solution (7.5 ml of 0.2 µm-filtered water and 2.5 mL of FBS preheated at 37 °C) was added drop by drop with a Pasteur pipette to the cell pellet. Cell suspension was incubated for 20 minutes at 37 °C. Tubes were in lean position for a better contact between the solution and the cells. Cells were centrifuged for 10 minutes at 1100 rpm. Cell pellets were washed and fixed with 3 successive baths of 10 mL ethanol/acetic acid (3:1) and stored at −20 °C in the last fixative solution until further use.

### FISH on chromosomes in metaphase

Suspensions of nuclei in metaphase stored at −20 °C were centrifuged, fixative solution was replaced by 2 mL of a fresh one. One mL of pipetted suspension was dropped on a clean Superfrost® glass slide (Thermo Scientific, Germany). The next day, slides were incubated for 1 hour in RNase solution containing 2X saline sodium citrate, SSC buffer (pH7, Biosolve, France) and 100 µg/mL of endoribonuclease A (Sigma-Aldrich, France). Slides were washed 3 times in 2X SSC buffer for 2 minutes at room temperature (RT) and dehydrated through successive 50%, 75%, 100% and 100% ethanol baths at RT. Nuclei on slides were denatured in 2X SSC buffer containing 70% of deionized formamide for 2 minutes at 72 °C. Simultaneously, orange 5′ TAMRA-TLR7, aqua X-centromere and green Y-centromere probes were denatured according to manufacturer’s instructions (Empire Genomics, United States). Slides were then washed 3 times for 2 minutes in 2X SSC buffer at 4 °C and dehydrated through successive 50%, 75% and 100% ethanol baths at 4 °C and a last 100% ethanol bath at RT. Probes were loaded onto the denatured slides and the hybridization area was sealed with 22 × 22 mm coverslips and rubber cement (Marabuwerke GmbH & Co, Germany). Slides were incubated overnight at 37 °C in a humid room. The following day, slides were washed 3 times with 0.5X SSC buffer (pH7) containing 0.1% SDS for 2 minutes. A droplet of Vectashield containing DAPI was added and slides were mounted for imagery using 22 × 60 mm coverslips. Images were visualized on a Zeiss AxioImager Apotom Z1 fluorescent microscope equipped with narrow band-pass filters for DAPI, CFP, FITC and DsRED fluorescence and an AxioCam MRm camera (Zeiss, Germany). A minimum of 1,000 nuclei were counted to assess *TLR7* extra-copies.

### Statistical analysis

Statistical analyses were conducted using GraphPad Prism 6 software (La Jolla, CA, USA). The non-parametric Mann-Whitney test was used to compare the distribution of relative *TLR7* and *TLR8* gene CN between patients and controls and to compare mRNA fold-regulation values between the two groups of patients (high or normal CN). For all correlation tested, Spearman’s rank test was used. *P*-values less than 0.05 were considered significant.

## Supplementary information

Supplementary figures
