# Supplementary material for: Mosaicism of XX and XXY cells accounts for high copy number of Toll like Receptor 7 and8 genes in peripheral blood of men with Rheumatoid Arthritis
Source: Sci Rep. 2019 Sep 9;9:12880. doi: 10.1038/s41598-019-49309-4 (PMC6733859; doi:10.1038/s41598-019-49309-4)
Supplement: Supplementary file 1 — Supplementary figures [file 41598_2019_49309_MOESM1_ESM.docx]

**Mosaicism of XX and XXY cells accounts for high copy number of *Toll like Receptor 7 and 8* genes in peripheral blood of men with Rheumatoid Arthritis.**

**Short Title: XX and XXY cells in men with Rheumatoid Arthritis**

Gabriel V. Martin^1,2#^, Sami B. Kanaan^1,2#^, Marie F. Hemon^1,2^ Doua F. Azzouz^1,2^, Marina El Haddad^1,2^, Nathalie Balandraud^1,3^, Cécile Mignon-Ravix^2,4^, Christophe Picard^5,6^, Fanny Arnoux^1,2^, Marielle Martin^1,2^, Jean Roudier^1,2,3^, Isabelle Auger^1,2^ and Nathalie C. Lambert ^1,2*^

^#^ G.V.M. and S.B.K. contributed equally to this study.

^1^INSERM UMRs1097, Parc scientifique de Luminy, Marseille, France ; ^2^Aix-Marseille Université, Marseille, France; ^3^Service de Rhumatologie, Hôpital Sainte Marguerite, AP-HM, Marseille, France ; ^4^Aix Marseille Univ, INSERM, MMG, Marseille, France; ^5^Centre National de la Recherche Scientifique (CNRS) UMR7268 (ADES), "Biologie des Groupes Sanguin", Marseille, France; ^6^Etablissement Français du Sang (EFS), Marseille, France

**SUPPLEMENTARY FIGURES**

**Supplementary Figure S1**. Increased copy number of *TLR7* and *TLR8* genes in blood samples from men with RA compared to age-matched healthy men. *TLR7* and *TLR8* gene copy numbers (CN) were calculated as detailed in the method section on peripheral blood DNA samples from men with RA (RA) age-matched one by one with healthy men (HM). P values are representative of Mann-Whitney test and indicate quantitative differences in CN between men with RA and HM. Grey filled squares represent individual men with RA and unfilled circles represent healthy men. On each plot, red bars represent mean values and standard deviations. An arbitrary threshold of high CN of *TLR7* and *TLR8* genes was defined as being superior or equal to the mean value observed in the 60 HM plus two standard deviations (≥1.11 for *TLR7* CN and ≥1.13 for *TLR8* CN represented by the blue dot line). Percentages of men with high CN are indicated in the upper section of the figure.

**Supplementary Figure S2**. Increased copy number of *TLR7* and *TLR8* genes in peripheral blood mononuclear cells (PBMC) from men with RA. *TLR7* and *TLR8* CN were evaluated on PBMC’s DNA samples from 62 men with RA and 35 healthy men as detailed in the method section. An increased CN of *TLR7* and *TLR8* genes was observed in men with RA compared to healthy men (Mann Whitney test). Grey filled squares represent individual men with RA and unfilled circles represent healthy men. On each plot, red bars represent mean values and standard deviations.

**
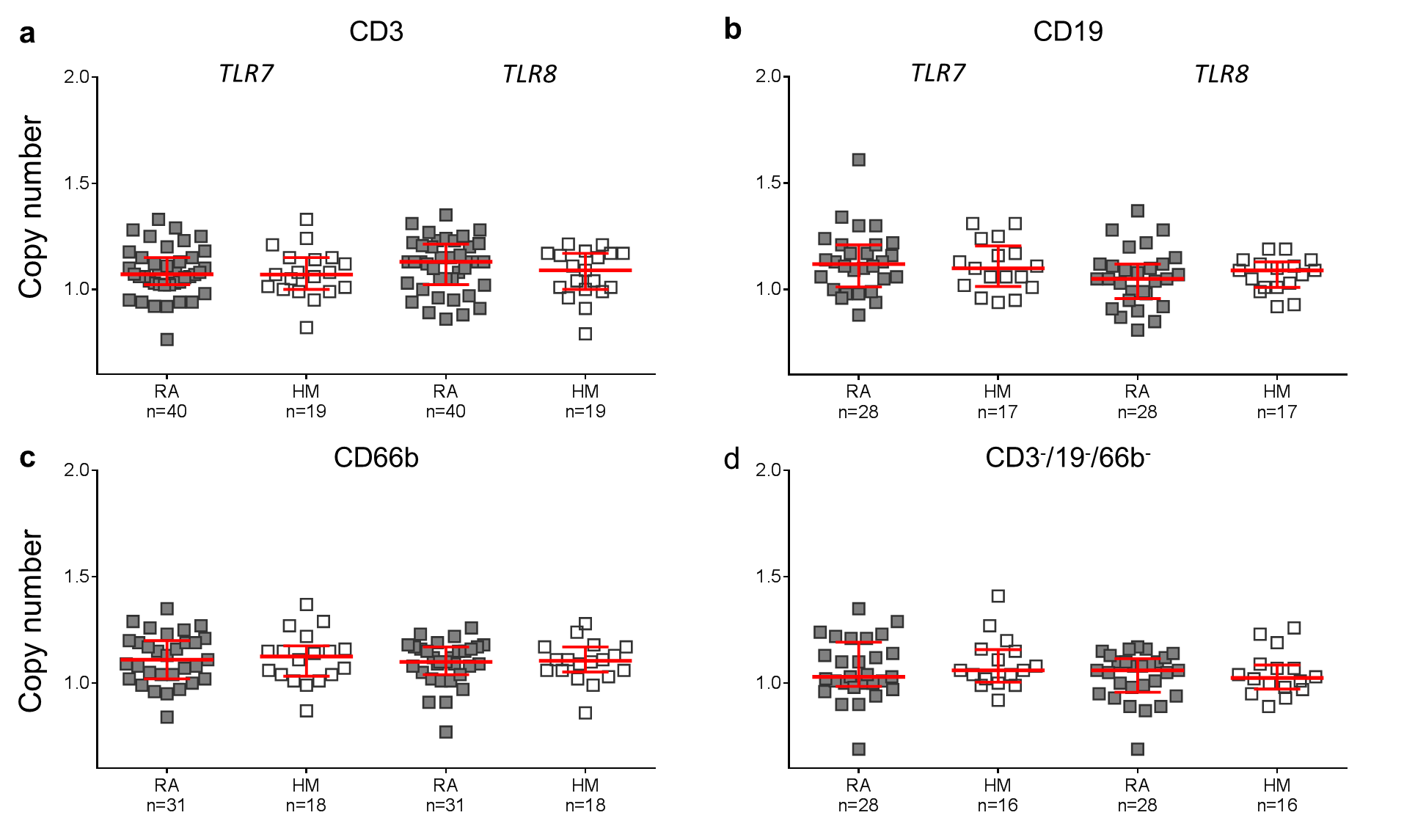
**

**Supplementary Figure S3.** No particular cell subpopulation being at increased *TLR7* or *TLR8* CN. Cells from peripheral blood were sorted, as indicated in the method section, into four subpopulations: **a** T cells (CD3 positive fraction), **b** B cells (CD19 positive fraction), **c** granulocytes (CD66b positive fraction) and **d** the depleted fraction of the former three (CD19^-^, CD3^-^, CD66b^-^ fraction), containing monocytes, macrophages, NK cells, and dendritic cells. On each plot, red bars represent the mean value and standard deviations.

**Supplementary Figure S4.** Repartition of XX nuclei and XXY nuclei in the 14 RA patients and 11 healthy controls selected to be tested by FISH. The number of XX or XXY nuclei counted per samples were standardized on 10,000 total nuclei. In red are represented XXY nuclei and in blue XX nuclei. Men with a high TLR7/8 gene copy number (z-score ≥ 2, i.e. being superior or equal to two standard deviations above the mean value observed in the healthy men) are mentioned with a black asterisk on the X axis.

***(a) (b)***

***(c) (d)***

***Supplementary Figure S5.*** Greater correlation between (**a**) TLR7 CN calculated by qPCR and the number of Klinefelter XXY nuclei observed by FISH (Spearman, R= 0.78, *P*<0.0001) rather than (**b**) the number of female XX nuclei (Spearman, R= 0.50, *P*=0.01). Similar results were observed with *TLR8* CN, respectively illustrated in (**c**) and (**d**) Results are from 14 men with RA (grey squares) and 11 healthy men (unfilled squares) simultaneously tested by qPCR and FISH.
